# Flexible lithium–oxygen battery based on a recoverable cathode

**DOI:** 10.1038/ncomms8892

**Published:** 2015-08-03

**Authors:** Qing-Chao Liu, Ji-Jing Xu, Dan Xu, Xin-Bo Zhang

**Affiliations:** 1 State Key Laboratory of Rare Earth Resource Utilization, Changchun Institute of Applied Chemistry, Chinese Academy of Sciences, Changchun 130022, China; 2 School of Materials Science and Engineering, Jilin University, Changchun 130012, China

## Abstract

Although flexible power sources are crucial for the realization next-generation flexible electronics, their application in such devices is hindered by their low theoretical energy density. Rechargeable lithium–oxygen (Li–O_2_) batteries can provide extremely high specific energies, while the conventional Li–O_2_ battery is bulky, inflexible and limited by the absence of effective components and an adjustable cell configuration. Here we show that a flexible Li–O_2_ battery can be fabricated using unique TiO_2_ nanowire arrays grown onto carbon textiles (NAs/CT) as a free-standing cathode and that superior electrochemical performances can be obtained even under stringent bending and twisting conditions. Furthermore, the TiO_2_ NAs/CT cathode features excellent recoverability, which significantly extends the cycle life of the Li–O_2_ battery and lowers its life cycle cost.

## Abstract (web summary)

Flexible energy storage systems usually have limited energy densities. Here the authors report a flexible lithium–oxygen battery with the cathode consisting of titanium dioxide nanowire arrays grown on carbon textiles, which displays high mechanical strength as well as promising electrochemical performance.

## Graphical abstract

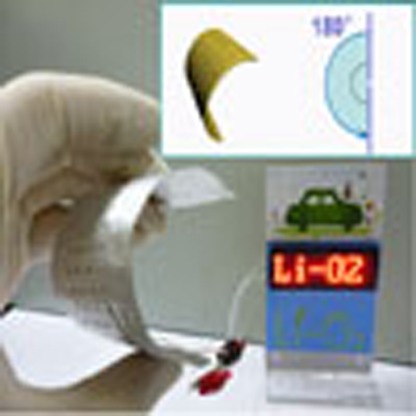

The worldwide demand for flexible electronics continues to grow rapidly because of their special advantages such as being lightweight, bendable, rugged, portable, rollable and potentially foldable, which have revolutionized several industries ranging from consumer products to the automotive, aerospace and medical industries^1^,^2^,^3^,^4^,^5^. To achieve a ‘flexible electronics' society, well-matched flexible energy storage/conversion devices are recognized as one of the key required components. However, current conventional power sources are too rigid and bulky to be integrated into flexible devices. Several inspirational prototypes have been developed in response including flexible lithium-ion batteries^6^,^7^,^8^,^9^,^10^, supercapacitors^11^,^12^,^13^,^14^,^15^ and solar cells^16^,^17^,^18^,^19^. However, the low theoretical energy density of these components intrinsically limits their application in next-generation flexible devices. Fortunately, rechargeable lithium–oxygen (Li–O_2_) batteries have emerged as one of the most promising electrochemical energy storage technologies because of their exceptionally high theoretical energy density of 3,600 Wh kg^−1^ (refs 20, 21, 22, 23, 24). Although the development of a flexible Li–O_2_ battery could theoretically meet the urgent demand for a high energy density battery in flexible devices, because this technology is still in its early stages, numerous scientific and technological challenges must first be overcome for the conventional Li–O_2_ battery, much less the flexible type. First, the carbon cathode is problematic because the decomposition of carbon material and its promotional effect on electrolyte decomposition leads to carbon cathode passivation and premature battery death^25^,^26^. Second, the highly conductive current collector, which includes metal foam and carbon paper, is inflexible. Finally, the cell configuration of conventional Li–O_2_ batteries (either the coin cell or Swagelok design) is exclusively packed with bulky and rigid stainless steel or engineering plastic. Therefore, there is an urgent need to first design and fabricate mechanically robust, electrochemically stable and highly effective components, as well as create a preferable cell configuration and structural design on the path toward flexible Li–O_2_ batteries.

We report a strategy to fabricate a flexible, free-standing and recoverable cathode by the seeds-assisted construction of unique hierarchical rutile TiO_2_ nanowire arrays (TiO_2_ NAs) grown onto carbon textiles (CT). A highly flexible Li–O_2_ battery with excellent mechanical strength and superior electrochemical performance, including a high round-trip efficiency, good rate capability and cycling stability, was fabricated by employing TiO_2_ NAs/CT as both a new class of cathode and current collector to replace the conventional rigid and bulky counterparts. Furthermore, we found that TiO_2_ NAs/CT exhibits excellent recoverability by simply rinsing after it loses function. This represents a new strategy to significantly extend the cycle life of Li–O_2_ batteries, which is still strongly limited by the instability of the electrolyte.

## Results

### Synthesis and analysis of TiO_2_ NAs grown onto CT

**Figure 1:**
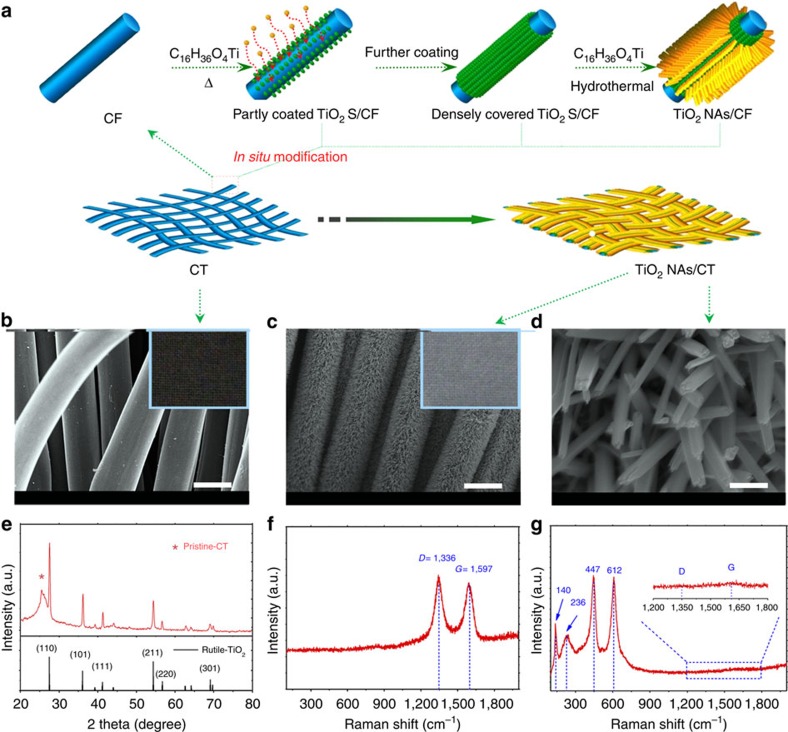
Scheme for fabrication and structure of the TiO_2_ NAs/CT cathode. (**a**) Schematic representations for the design and preparation of the TiO_2_ NAs/CT. (**b**) Scanning electron microscope image and photograph (inset) of pristine–CT (scale bar, 10 μm). (**c**) SEM image and photograph (inset) of the obtained TiO_2_ NAs/CT cathode (scale bar, 10 μm). (**d**) Enlarged image of **c** with 500-nm scale bars. (**e**) X-ray diffraction patterns of the obtained TiO_2_ NAs/CT cathode. The asterisk is indexed to carbon textiles. (**f**) Raman spectra of the pristine–CT cathode. (**g**) Raman spectra of the TiO_2_ NAs/CT cathode.

Figure 1a shows the synthesis strategy for producing TiO_2_ NAs/CT. First, to protect the CT surface effectively, the TiO_2_ seeds are deposited densely and homogenously onto the carbon fibre of the CT. Then, to accommodate more discharge products in the Li–O_2_ cell, the TiO_2_ NAs are grown *in situ* by the TiO_2_ seeds-directed coordination self-assembly method. Scanning electron microscopic (SEM) images show that the pristine–CT is woven by carbon fibre with diameter of ca. 10 μm (Fig. 1b) and that the TiO_2_ NAs are vertically and homogenously grown onto the skeleton of the CT without the help of any additional binder or solvent (Fig. 1c,d). This ensures the formation of a free-standing structure and a favourable low-resistance pathway for electron transportation. The average diameter of the nanorods is ca. 50 nm (Supplementary Fig. 1a). The high-resolution transmission electron microscopy image collected at the surface of the TiO_2_ NAs reveals lattice fringes of 0.32 nm in the (110) planes, corresponding to the rutile TiO_2_ phase (Supplementary Fig. 1b), which were also confirmed by X-ray diffraction (Fig. 1e). Our measured Raman spectra reveal that both the D- (1,336 cm^−1^) and G-band (1,597 cm^−1^) of the pristine–CT nearly vanish from the TiO_2_ NAs/CT sample (Fig. 1f,g), demonstrating the effective protection of the CT surface by TiO_2_ NAs^16^. The SEM image of TiO_2_ NAs/CT and the corresponding elemental mapping images of C, O and Ti demonstrate the core-shell configuration of TiO_2_ NAs/CT (Supplementary Fig. 2). More importantly, the obtained TiO_2_ NAs/CT cathode is high flexible (Supplementary Fig. 3). Compared with its traditional counterpart, this novel cathode features several tailored properties. First, the TiO_2_ NAs/CT as a free-standing cathode without any polymeric binder can facilitate the high flux of electron transportation throughout the cathode and also avoid the parasitic reaction caused by non-conductive polymeric binder^27^. Second, the TiO_2_ NAs/CT cathode effectively avoids a series of issues caused by the carbon cathode, as described in many other reports, such as the degradation of carbon itself and electrolyte decomposition promoted by carbon material^25^. Finally, the TiO_2_ NAs/CT is flexible, which is crucial for the assembly of a flexible Li–O_2_ battery. All of these advantages benefit the electrochemical performance of the flexible Li–O_2_ battery.

### Properties of the TiO_2_ NAs/CT cathode

**Figure 2:**
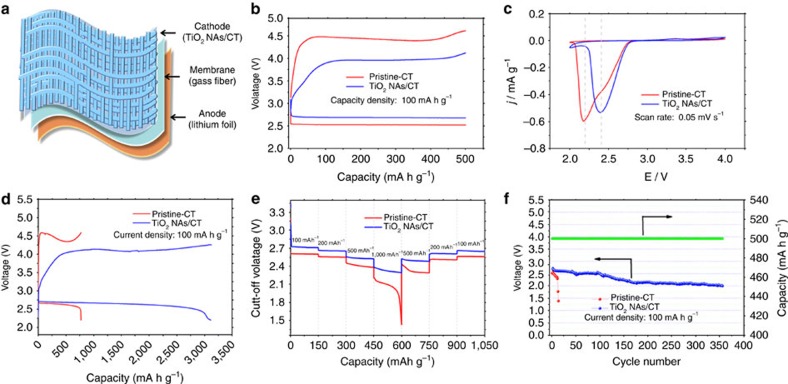
Cell structure and electrochemical performance. (**a**) Schematic illustration of the cell assembly composed of TiO_2_ NAs/CT (cathode), glass fibre (separator) and lithium foil (anode). (**b**) First discharge–charge curves of the Li–O_2_ cells with a pristine–CT cathode and a TiO_2_ NAs/CT cathode at a current density of 100 mA g^−1^.The specific capacity was limited to 500 mAh g^−1^. (**c**) CVs of Li–O_2_ cells with the two types of cathodes at a constant scan rate of 0.05 mV s^−1^. (**d**) Full range test of the Li–O_2_ cells with a pristine–CT cathode and a TiO_2_ NAs/CT cathode at a current density of 100 mA g^−1^. These tested cathodes were discharged with the cutoff voltage limited to 2.2 V and then recharged with the equivalent discharge capacity. (**e**) The rate capability of the Li–O_2_ cells with the two types of cathodes at different current densities. (**f**) Voltage versus cycle number on the discharge terminal of the Li–O_2_ cell with a TiO_2_ NAs/CT cathode.

We constructed a flexible Li–O_2_ battery device that contained a flexible pristine–CT or TiO_2_ NAs/CT cathode, a glass fibre separator, and a lithium foil anode (Fig. 2a). Lithium triflate (LiCF_3_SO_3_) in tetraethylene glycol dimethyl ether (TEGDME) was employed as the electrolyte because of its reported relatively high stability towards superoxide (O_2_^−^) (refs 28, 29, 30). The first discharge and charge voltage of the Li–O_2_ battery can be significantly improved with the help of the TiO_2_ NAs/CT cathode. This cathode enhances the round-trip efficiency, which is vital for electrochemical energy storage devices (Fig. 2b). Specifically, compared with the Li–O_2_ cell with a pristine–CT cathode, the discharge voltage and charge voltage of that with a TiO_2_ NAs/CT cathode is higher by 160 mV and lower by 495 mV, respectively. This result is further supported by the cyclic voltammograms of Li–O_2_ cells with either a pristine–CT or TiO_2_ NAs/CT cathode (Fig. 2c). The cell with a TiO_2_ NAs/CT cathode has a higher peak potential compared with the cell with a pristine–CT cathode, suggesting that TiO_2_ NAs/CT exhibits better ORR catalytic activity. However, the peak current of the TiO_2_ NAs/CT cathode is lower than that of the pristine–CT cathode, which might be due to the lower conductivity of TiO_2_ compared with that of pristine–CT. In addition, the Li–O_2_ cell with a TiO_2_ NAs/CT cathode exhibits a much higher discharge capacity than that with a pristine–CT cathode (3,000 mAh g^−1^ versus 770 mAh g^−1^) (Fig. 2d). Furthermore, to exclude possible electrochemical contributions from the intercalation of lithium ions (Li^+^) into pristine–CT or TiO_2_ NAs/CT materials, the initial discharge curves of Li–O_2_ cells with pristine–CT and TiO_2_ NAs/CT cathodes under an argon (Ar) atmosphere were also obtained for comparison (Supplementary Fig. 4). Clearly, the background discharge capacity is negligible within the voltage range, which suggests that the above obtained enhanced discharge capacities of the Li–O_2_ cells are derived from the oxygen reduction. Our rate performance investigations show that the discharge voltage plateau of the TiO_2_ NAs/CT cathode is higher than that of pristine–CT at each current density (Fig. 2e, consistent with Fig. 2b). Furthermore, the cell with the TiO_2_ NAs/CT cathode can discharge/charge for >356 cycles with a discharge terminal voltage >2.0 V, which is ca. 30 times longer than the cell with the pristine–CT cathode, that is, 12 cycles (Fig. 2f, Supplementary Fig. 5). All of these improvements, including the cyclic stability with the capacity limit of 1,000 mAh g^−1^ (Supplementary Fig. 6), further confirm the advantages of the TiO_2_ NAs/CT cathode, which may be attributed to the synergistic effect of the high catalytic activity and the tailored free-standing structure of the TiO_2_ NAs/CT cathode. TiO_2_ NAs provides enough void volume for the deposition of discharge products, therefore, resulting in a much enhanced discharge capacity. In general, although charge transfer is the rate-determining process at low current densities, when the current density increases, the mass transfer of lithium ions and oxygen can become the rate-determining process. A uniform oxygen and lithium-ion distribution inside the TiO_2_ NAs/CT cathode is required to improve the rate capability, especially, at very high current densities^31^. Furthermore, the morphology and crystallinity of the discharge product in TiO_2_ NAs/CT cathode also contributes positively to the high specific capacity, rate capability and cycling stability of Li–O_2_ cells (*vide infra*).

### Bending and twisting properties

**Figure 3:**
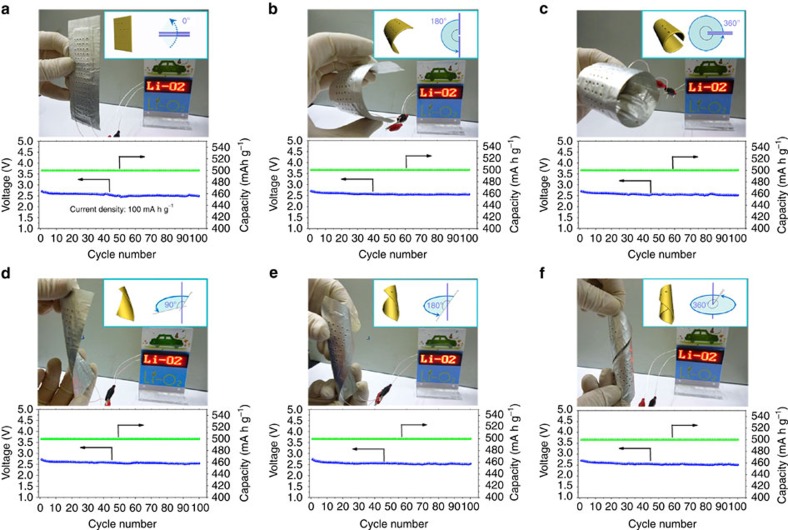
The bending and twisting properties of the Li–O_2_ battery device. (**a**–**c**) The bending properties with the device bent to 0°, 180° and 360°, respectively. (**d**–**f**) The twisting properties with the device twisted to 90°, 180° and 360°, respectively. The corresponding variation of terminal discharge voltage versus cycle number of the Li–O_2_ cells with TiO_2_ NAs/CT cathode is shown in each panel.

To demonstrate its potential application in flexible electronics, the as-fabricated flexible Li–O_2_ battery with a TiO_2_ NAs/CT cathode was used to power a commercial red light-emitting diode display screen (Fig. 3). The terminal discharge voltage of the devices after being bent to 180° (Fig. 3b) and 360° (Fig. 3c) were 2.55 and 2.52 V after 100 cycles, respectively, which was even slightly better than that without bending (2.49 V, Fig. 3a). Even under more stringent conditions, the terminal discharge voltages after 100 cycles were 2.54, 2.51 and 2.52 V at torsion angles of 90° (Fig. 3d), 180° (Fig. 3e) and 360° (Fig. 3f), respectively. Furthermore, it can be seen that the terminal discharge–charge voltage versus cycle number of the device remained almost constant even after 100 cycles, revealing that the electrochemical stability of the fabricated flexible Li–O_2_ battery is hardly affected by external bending or twisting strains (Supplementary Fig. 7). In addition, the TiO_2_ NAs/CT cathode also possesses good mechanical integrity even after twisting for 1,000 cycles (Supplementary Fig. 8). These results collectively demonstrate the excellent flexibility of this newly constructed flexible Li–O_2_ device, and no structural failure was observed after various bending and twisting tests.

### Analysis of the discharge products

**Figure 4:**
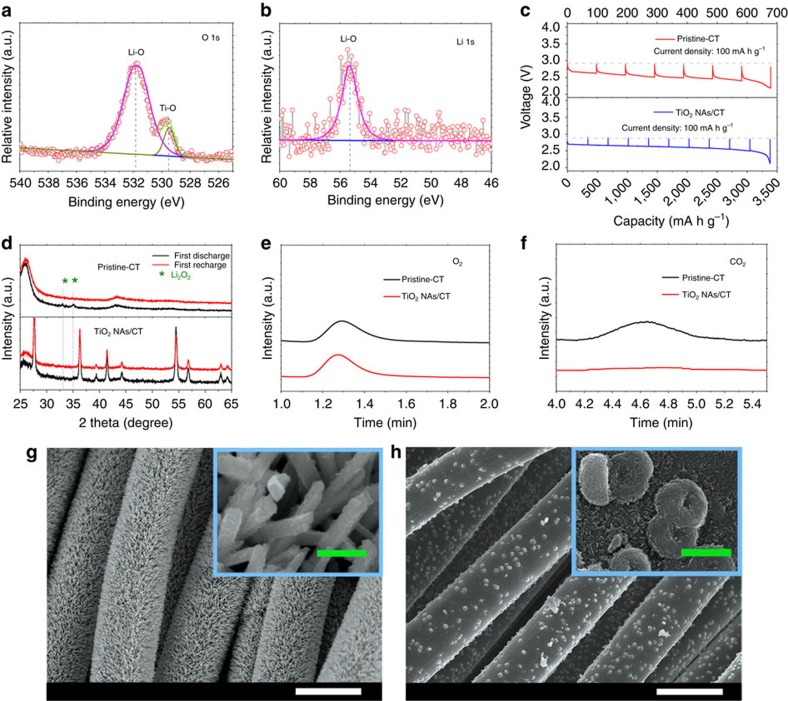
Characterization of discharge products. (**a**) O1s X-ray photoelectron spectroscopy (XPS) spectra of the discharged TiO_2_ NAs/CT cathode. (**b**) Li1s XPS spectra of the discharged TiO_2_ NAs/CT cathode. (**c**) GITT discharge voltage profile obtained from the Li–O_2_ cell with a pristine–CT cathode (red) and (**a**) TiO_2_ NAs/CT cathode (blue) at first discharge with a current density of 100 mA g^−1^. Both curves show an equilibrium potential of the Li–O_2_ cell of near 2.9 V regardless of the state of discharge, which is in accordance with the formation potential of Li_2_O_2_. (**d**) Corresponding X-ray diffraction patterns of the two types of discharged cathodes. (**e**) Gas chromatography (GC) signals of O_2_ released after charging the Li–O_2_ cells with a pristine–CT cathode (black) and a TiO_2_ NAs/CT cathode (red), which were obtained with a thermal conductivity detector (TCD). (**f**) GC signal of CO_2_ released. (**g**) SEM images of the discharged TiO_2_ NAs/CT cathode with a current density of 100 mA g^−1^ (white scale bar, 10 μm, green scale bars: 500 nm). (**h**) SEM images of the discharged pristine–CT cathode with a current density of 100 mA g^−1^ (white scale bar, 10 μm, green scale bar, 500 nm).

The discharge products of the Li–O_2_ battery with a TiO_2_ NAs/CT cathode were investigated using X-ray photoelectron spectroscopy (Fig. 4a,b) The peaks positioned at 55.5 and 531.9 eV can be assigned to the Li–O bond of lithium peroxides (Li_2_O_2_)^32^, which is supported by the equilibrium potential of the Li–O_2_ battery of ca. 2.9 V (theoretical formation potential of Li_2_O_2_) obtained using the galvanostatic intermittent titration technique (Fig. 4c)^33^. However, when compared with X-ray diffraction patterns of the discharged pristine–CT cathode (Fig. 4d), the characteristic peaks of Li_2_O_2_ were not observed in the TiO_2_ NAs/CT cathode, demonstrating the amorphous nature of Li_2_O_2_ formed in the TiO_2_ NAs/CT cathode. It has been reported that TiO_2_ NAs may possess a suitable oxygen binding energy and high affinity for oxygen coverage. This would lead to stronger oxygen adsorption on the TiO_2_ NAs surface than that of the CT, which may facilitate the formation of amorphous Li_2_O_2_ (ref. 34). Furthermore, in sharp contrast to the conventional toroidal morphology of the discharge product obtained on the pristine–CT cathode (Fig. 4h)^35^,^36^, amorphous Li_2_O_2_ films are homogenously coated onto TiO_2_ NAs (Fig. 4g). This amorphous Li_2_O_2_ film may contain many defects (for example, lithium vacancies) that provide channels for electron and especially ion conduction, thus enhancing the electrode kinetics during the charge process towards a reduced charge overpotential (Fig. 2b)^37^,^38^. In addition, it should be noted that recent theoretical calculations have demonstrated that polycrystalline grain boundaries of Li_2_O_2_ can also significantly influence the charging overpotential of a Li–O_2_ battery^39^. Therefore, because Li–O_2_ batteries are still relatively new, additional research efforts including *in situ* transmission electron microscopy (TEM) observations should be devoted to clarifying the effect of the crystallinity of Li_2_O_2_ on the charging process of Li–O_2_ batteries. Although the toroidal products decorated on the CT are mostly decomposed after subsequent recharging processes (Supplementary Fig. 9a), the surface of CT becomes rough and many holes appear on the pristine–CT cathode (Supplementary Fig. 9b) compared with the original one (Fig. 1b). This indicates the existence of serious parasitic reactions between the CT and Li_2_O_2_ and/or nascent O_2_ (ref. 31), which was confirmed by the large amount of CO_2_ gas generated (2.12 μl) during the charging process (Supplementary Figs 10 and 11, and Supplementary Table 1). In sharp contrast, for the TiO_2_ NAs/CT cathode, the TiO_2_ NAs recovered to its initial state, with the surface becoming smooth again (Supplementary Fig. 9d), which was confirmed by a trace amount of CO_2_ gas generated (0.04 μl; Fig. 4f, Supplementary Fig. 12, Supplementary Table 1), wherein the reaction mechanism during discharge/charge processes are proposed to be investigated by *in situ* differential electrochemical mass spectroscopy in the future. Unexpectedly, the smooth surface of the TiO_2_ NAs/CT cathode was retained even after multiple cycles (10th, 50th and 100th cycle) of the Li–O_2_ cell, which demonstrates the good rechargeability of the TiO_2_ NAs/CT cathode and the stability of TiO_2_ NAs (Supplementary Figs 13 and 14).

## Discussion

**Figure 5:**
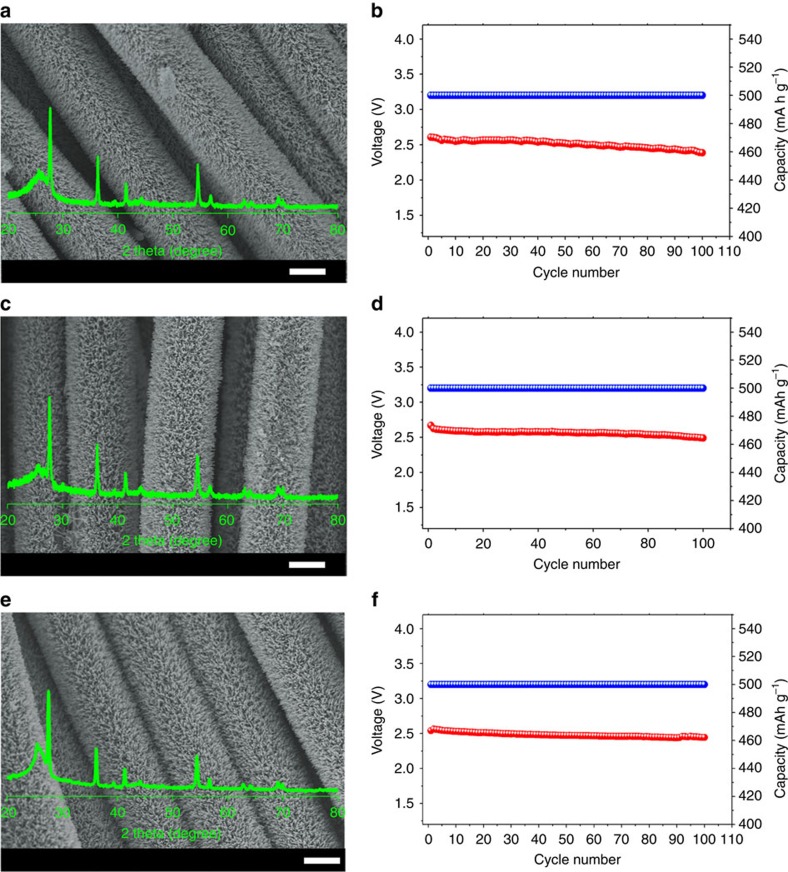
Recoverable performance. (**a**) SEM images of the pristine TiO_2_ NAs/CT cathode. (scale bar, 5 μm). The inset in **a** is the corresponding X-ray diffraction pattern. (**b**) The variation of the terminal discharge voltage of the pristine TiO_2_ NAs/CT cathode with a current density of 100 mA g^−1^. (**c**) SEM images of the first-recovered TiO_2_ NAs/CT cathode (scale bar, 5 μm). The inset in **c** is the corresponding X-ray diffraction pattern. (**d**) The variation of the terminal discharge voltage of the first-recovered TiO_2_ NAs/CT cathode with a current density of 100 mA g^−1^. (**e**) SEM images of the 10th-recovered TiO_2_ NAs/CT cathode (scale bar, 5 μm). The inset in **e** is the corresponding X-ray diffraction pattern. (**f**) The variation of the terminal discharge voltage of the tenth recovered TiO_2_ NAs/CT cathode with a current density of 100 mA g^−1^.

Electrolyte decomposition, which is due to the continuous accumulation of the side products such as Li_2_CO_3_ and Li alkyl carbonates on the cathode surface (Supplementary Fig. 15), still inevitably resulted in premature death of the Li–O_2_ battery. Currently, these cathodes can only be abandoned due to serious damage of the structure and chemical composition. Inspired by the rather high structural and chemical stability of the TiO_2_ NAs/CT cathode, we simply rinsed the TiO_2_ NAs/CT cathode (even after 100 discharge/charge cycles) with 2 M HCl to remove the residual carbonates. Interestingly, the free-standing structure and chemical composition of the disabled TiO_2_ NAs/CT cathode were restored after washing, demonstrating the excellent recoverability of the TiO_2_ NAs/CT cathode (Fig. 5c). Unexpectedly, the rebuilt cell exhibits almost the same voltage profiles (Fig. 5d) as the fresh one (Fig. 5b), even after 100 cycles. To further demonstrate its superior recoverability, the 3rd-, 5th-, and 10th-recovered TiO_2_ NAs/CT cathode was also investigated, as shown in Fig. 5e,f and Supplementary Fig. 16. Although the TiO_2_ NAs/CT cathode was recovered 10 times, there was no obvious degradation of the structure or electrochemical performance of the TiO_2_ NAs/CT cathode (Fig. 5e,f).

In conclusion, we have shown that a highly flexible Li–O_2_ battery can be constructed by employing a free-standing and recoverable TiO_2_ NAs/CT cathode. Superior electrochemical performances in terms of round-trip efficiency, rate capability and cycling stability, even under harsh bending and twisting conditions, have been achieved. These characteristics may be attributable to the tailored properties of the TiO_2_ NAs/CT cathode, which include high mechanical and chemical stability as well as high catalytic activity. Furthermore, the excellent recoverability of the TiO_2_ NAs/CT cathode can significantly extend the cycle life (at least 1,000 cycles) and decrease the whole-life cycle cost of Li–O_2_ batteries. However, if the same benefits could be extended to a more highly efficient cathode (Supplementary Figs 17–19) and coupled with a next-generation stable electrolyte, an advanced flexible Li–O_2_ battery could be expected to dominate the upcoming field of flexible electronics. Hence, the results obtained here will hopefully encourage further studies on flexible Li–O_2_ cells, although numerous challenges precluding their use in practical devices remain.

## Methods

### Chemicals and materials

Titanium *n*-butoxide (C_16_H_36_O_4_Ti), isopropanol (C_3_H_8_O), ethanol (EtOH), hydrochloric acid (HCl) and acetone (C_3_H_6_O) were purchased from Sinopharm Chemical Reagent Co. Ltd., Shanghai, China. Tetraethylene glycol dimethyl ether, lithium triflate (LiCF_3_SO_3_) and sodium tetrachloropalladate (Na_2_PdCl_4_) were purchased from Aladdin Reagent. Carbon textiles were purchased from Torray.

### TiO_2_ NAs/CT cathode preparation

The TiO_2_ NAs/CT cathode was synthesized using a seed-assisted method. The CT were ultrasonically cleaned with C_3_H_6_O and distilled water several times and then dried at 60 °C in a vacuum oven. The clean CT were immersed in a 0.075 M titanium *n*-butoxide isopropanol solution, rinsed with ethanol, and then dried in an air oven at 60 °C three times. The dried CT were subsequently heated in air at 500 °C for 1 h, forming TiO_2_ nanoparticles coated on the CT. Next, 0.66 ml titanium *n*-butoxide isopropanol was added into a solution of 15 ml concentrated hydrochloric acid and 15 ml deionized water. The mixture was stirred for >6 h and a clear solution was obtained. This clear solution together with the CT-coated TiO_2_ nanoparticles were transferred to a Teflon-lined stainless steel autoclave (50 ml volume) and heated in an oven at 150 °C for 12 h. When the oven cooled down, the CT were removed from the autoclave and rinsed with deionized water several times and dried in an air oven at 60 °C. Finally, the sample was annealed in air at 550 °C for 2 h.

### Fabrication of Pd decorated TiO_2_ NAs/CT cathode

The obtained TiO_2_ NAs/CT cathode was directly immersed into Na_2_PdC_l4_ aqueous solution (5 mM) and exposed to light for 30 min. The cathode was finally rinsed with deionized water several times and dried in an air oven at 60 °C.

### Characterization

The morphologies and structures of the materials were characterized using various physiochemical techniques, including X-ray diffraction, field emission scanning electron microscopy, TEM, and Raman spectral analysis. The discharge and recharge products were characterized using X-ray photoelectron spectroscopy , X-ray diffraction, gas chromatography and mass spectrometry.

### Assembling of the flexible Li–O_2_ battery device

The flexible Li–O_2_ battery device was assembled in an argon-filled glove box using a commercial lithium belt anode, a glass fibre separator, an oxygen cathode and a 1-M LiCF_3_SO_3_ in TEGDME electrolyte. The as-fabricated TiO_2_ NAs/CT (6 × 2 cm) was directly used as the air cathode without any polymeric binder.

### Recovery of the TiO_2_ NAs/CT cathode

TiO_2_ NAs/CT cathode was disassembled from the Li–O_2_ battery device after multiple cycles, rinsed with 2 M HCl, washed with distilled water several times and subsequently dried in air oven at 60 °C for utilization.

### Instrumentation

SEM was performed on a Hitachi S-4800 field emission scanning electron microscope operating with an acceleration voltage of 10 kV. Samples for SEM were prepared by directly placing the electrode sample onto an SEM brass stub. The X-ray diffraction measurements were performed on a Bruker D8 Focus X-ray diffractometer using Cu *Kα* radiation. TEM was performed using an FEI Tecnai G2 S-Twin transmission electron microscope with a field emission gun operating at 200 kV. The X-ray photoelectron spectroscopy measurements were performed on an ESCA-LAB 250 photoelectron spectrometer. Electrochemical impedance spectroscopy and CV measurements were performed on a BioLogic VMP3 electrochemical workstation. The Li–O_2_ battery device measurements were cycled on a LAND CT2001A multichannel battery testing system. For the gas chromatography (Techcomp GC-7900) measurements, a glass chamber containing the Li–O_2_ battery following discharging was flooded with Ar. The gas was then collected for analyses after the battery was charged. O_2_ and CO_2_ were analysed with a thermal conductivity detector using Ar as the carrier gas (detection limit: 10 p.p.m.). Mass analysis of the generated gases was performed using an OmniStar GSD 320 system (Pfeiffer Vacuum) mass spectrometry, wherein argon gas was chosen as the carrier gas. A xenon light source (PLS-SXE300C) was employed for the photochemical deposition of Pd nanoparticles.

## Additional information

**How to cite this article:** Liu, Q.-C. *et al*. Flexible lithium–oxygen battery based on a recoverable cathode. *Nat. Commun*. 6:7892 doi: 10.1038/ncomms8892 (2015).

## Supplementary Material

Supplementary Information Supplementary Figures 1-19, Supplementary Table 1 and Supplementary Reference
