# Supplementary material for: Flexible lithium–oxygen battery based on a recoverable cathode
Source: Nat Commun. 2015 Aug 3;6:7892. doi: 10.1038/ncomms8892 (PMC4532833; doi:10.1038/ncomms8892)
Supplement: Supplementary Information — Supplementary Figures 1-19, Supplementary Table 1 and Supplementary Reference [file ncomms8892-s1.pdf]

Supplementary Figures

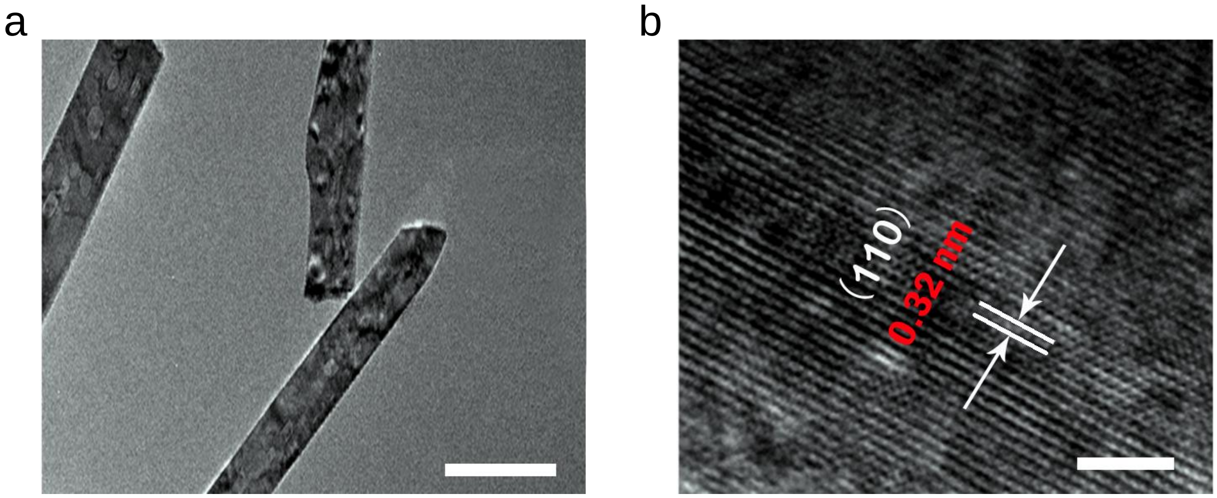

Supplementary Figure 1 | Structure of the TiO_2 NAs/CT cathode. (**a**) Transmission electron microscopy (TEM) image of the TiO_2 NAs. Scale bars, 100 nm. (**b**) HRTEM image of (**a**). Scale bars, 2 nm.

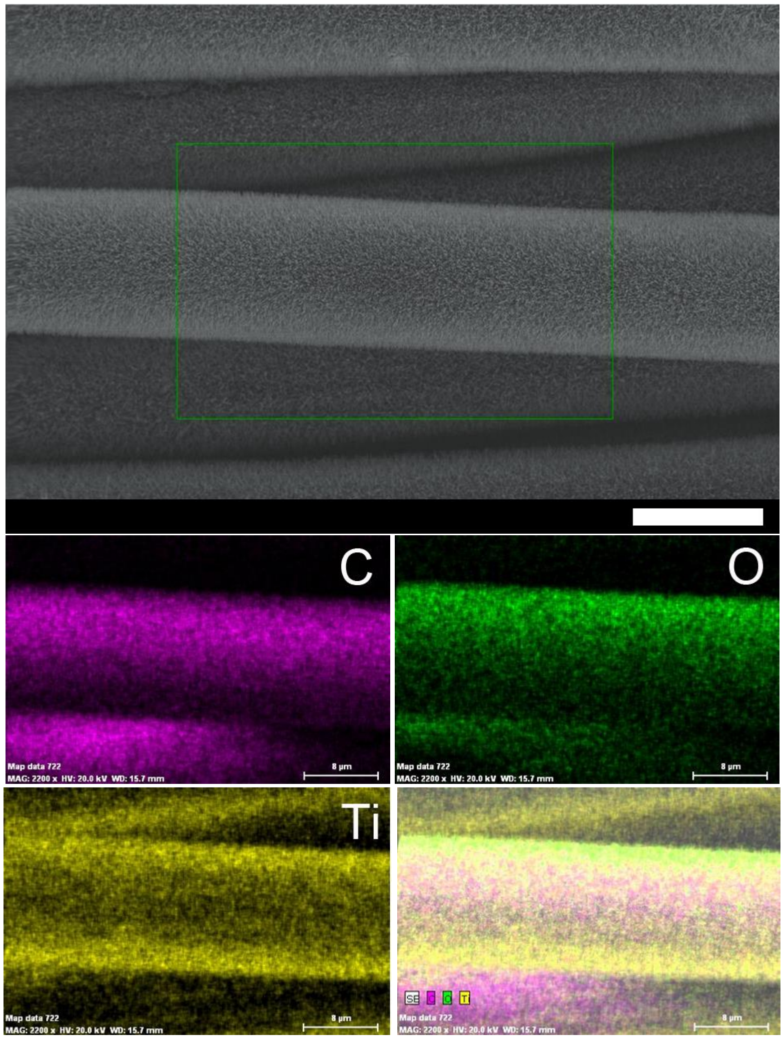

Supplementary Figure 2 | SEM mapping images. SEM image of TiO_2 NAs/CT and the corresponding elemental mapping images of C, O and Ti and all three elements in the composite. Scale bars, 10 µm. The C element is distributed with diameter of 12 µm. The O and Ti elements are uniformly distributed around a single CT. The diameter of the resulting composite TiO_2 NAs/CT had increased to about 13 µm from 12 µm in the pristine-CT. All these demonstrate the core-shell configuration of TiO_2 NAs/CT and this structure could effectively prevent the pristine-CT from decomposing.

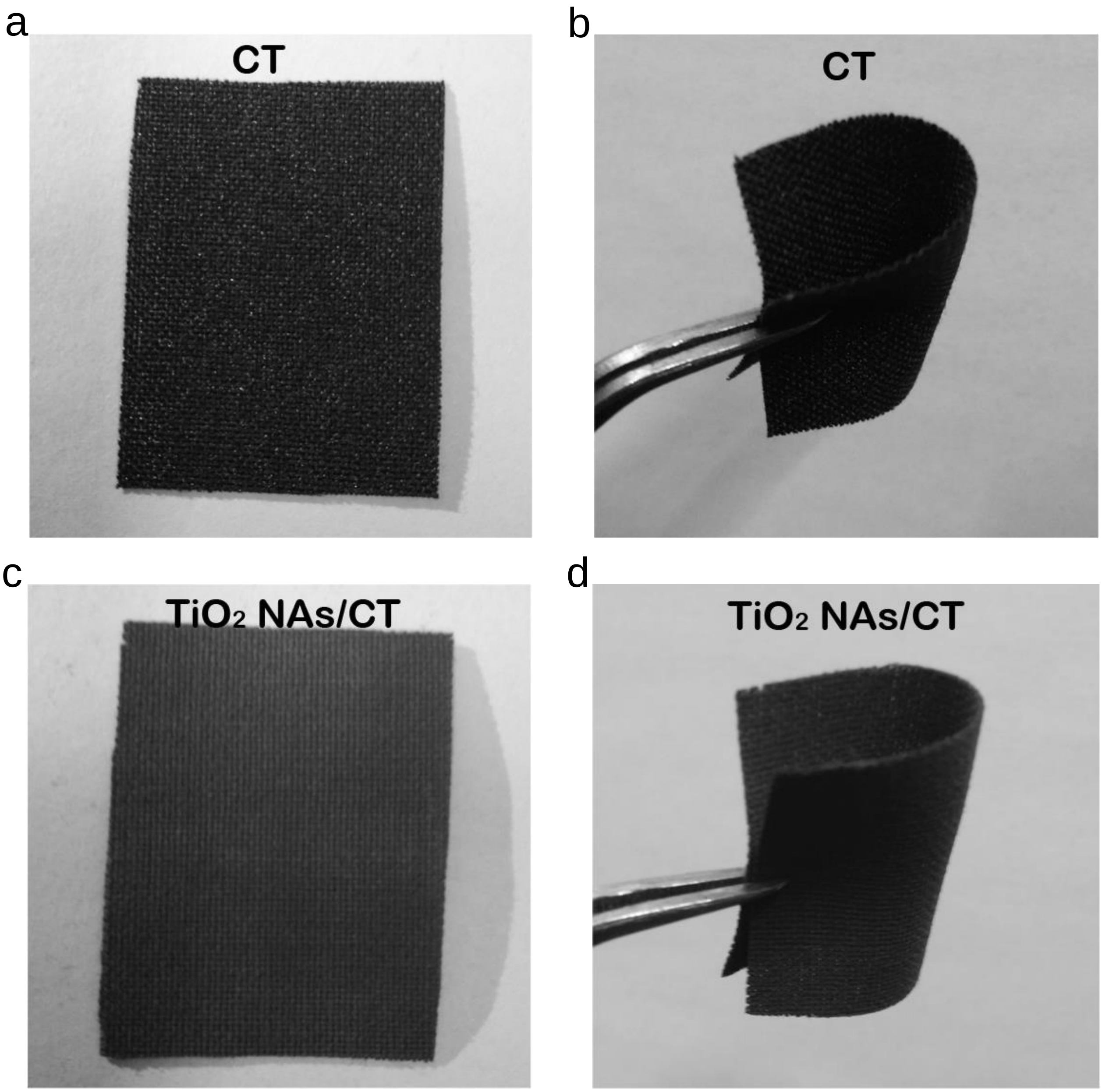

Supplementary Figure 3 | Optical photographs. Optical photographs of (**a, b**) CT and (**c, d**) TiO_2 NAs/CT cathodes.

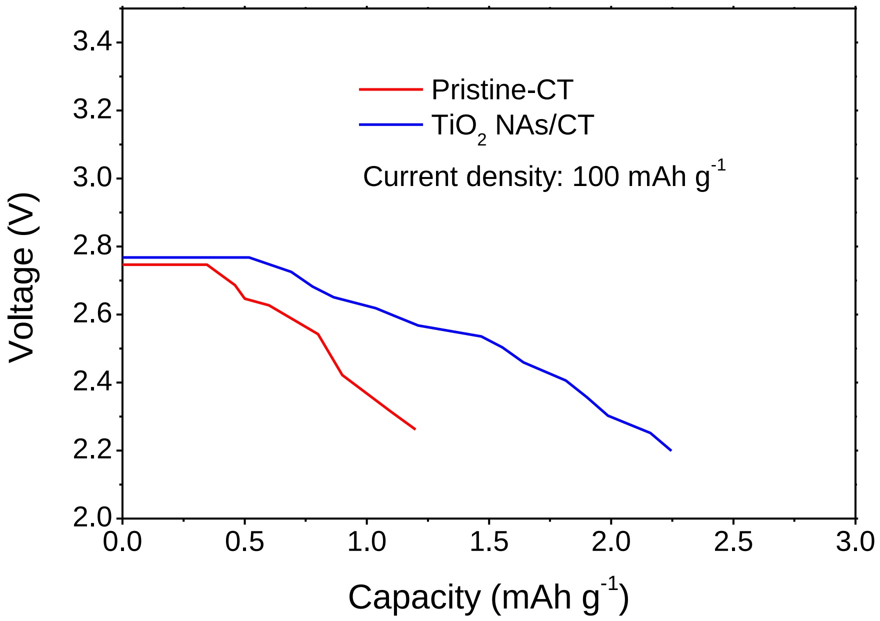

Supplementary Figure 4 | Discharge curves of the Ar-filled cells. The current density, 100 mA g^{-1}. The background discharge capacity is negligible within the voltage range, which suggests that the discharge capacities of Li-O_2 cells are derived from the oxygen reduction.

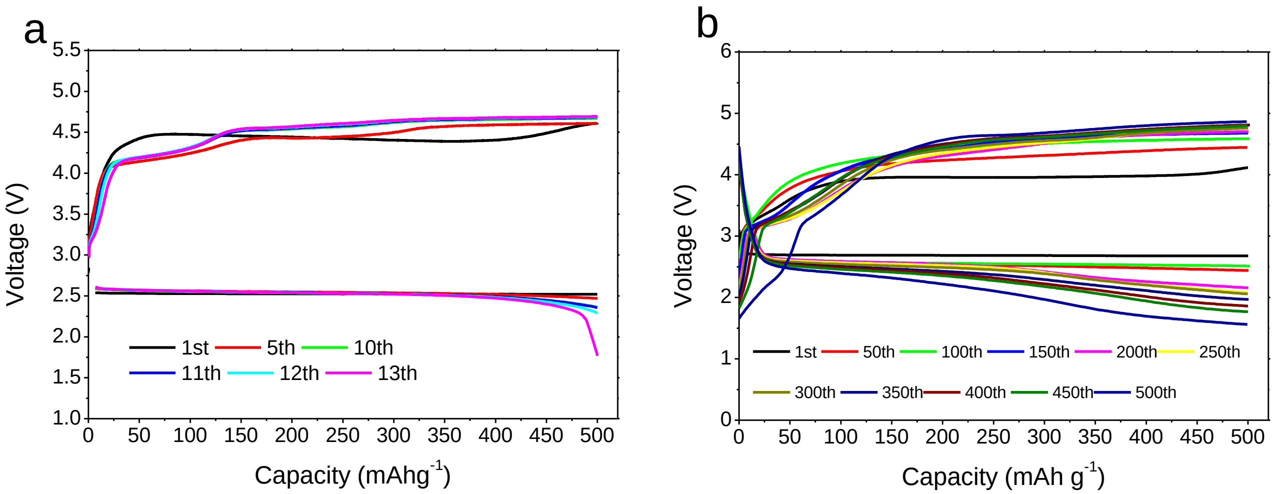

Supplementary Figure 5 | Cycling performance. Discharge-charge curves of the Li-O_2 cells with the (**a**) pristine-CT and (**b**) TiO_2 NAs/CT cathodes at different cycles. Current density: 100 mA g^{-1}.

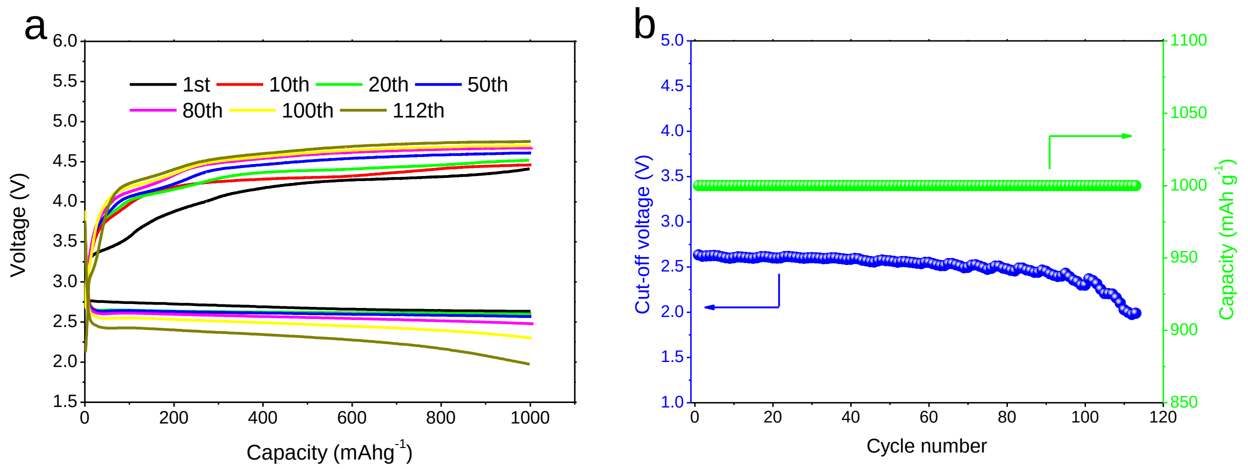

Supplementary Figure 6 | Cycling performance. Discharge-charge curves of flexible Li-O_2 cells with TiO_2 NAs/CT cathode (**a**) and corresponding cycling performance (**b**) with a fix capacity of 1000 mAh g^{-1} at a current density of 100 mA g^{-1}.

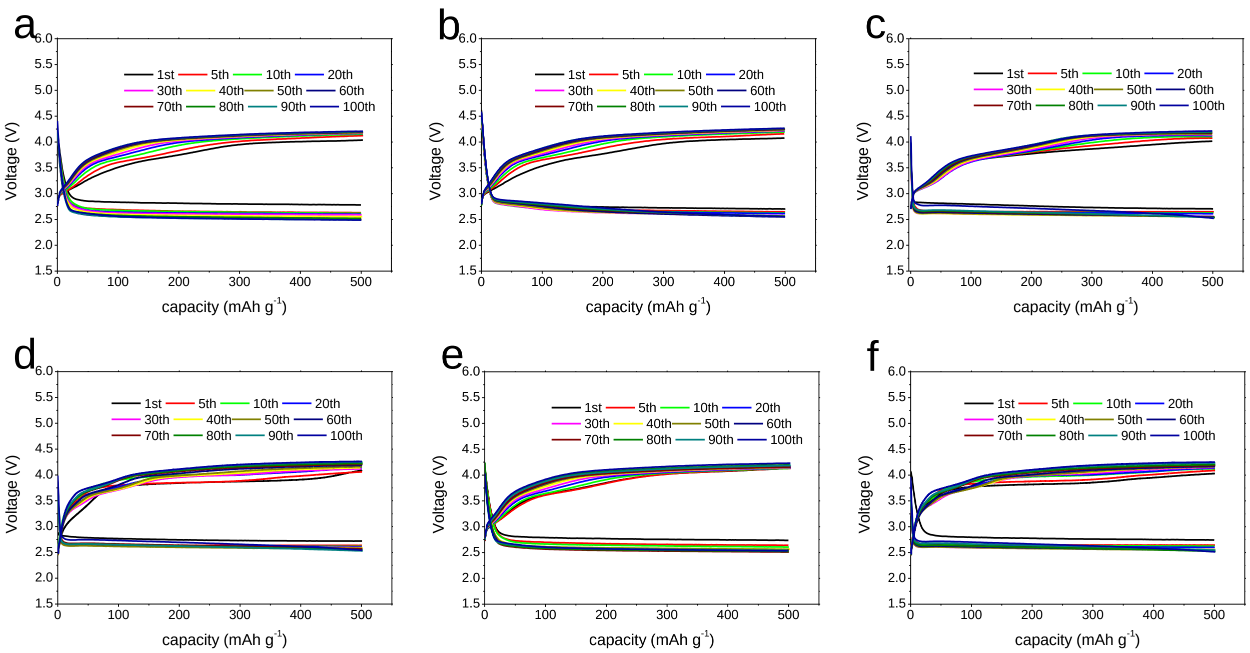

Supplementary Figure 7 | Cycling performance. (**a-c**) Discharge-charge curves of the Li-O_2 cells with the devices bended to 0°, 180° and 360°, respectively. (**d-f**) Discharge-charge curves of the Li-O_2 cells with the devices twisted to 0°, 180° and 360°, respectively.

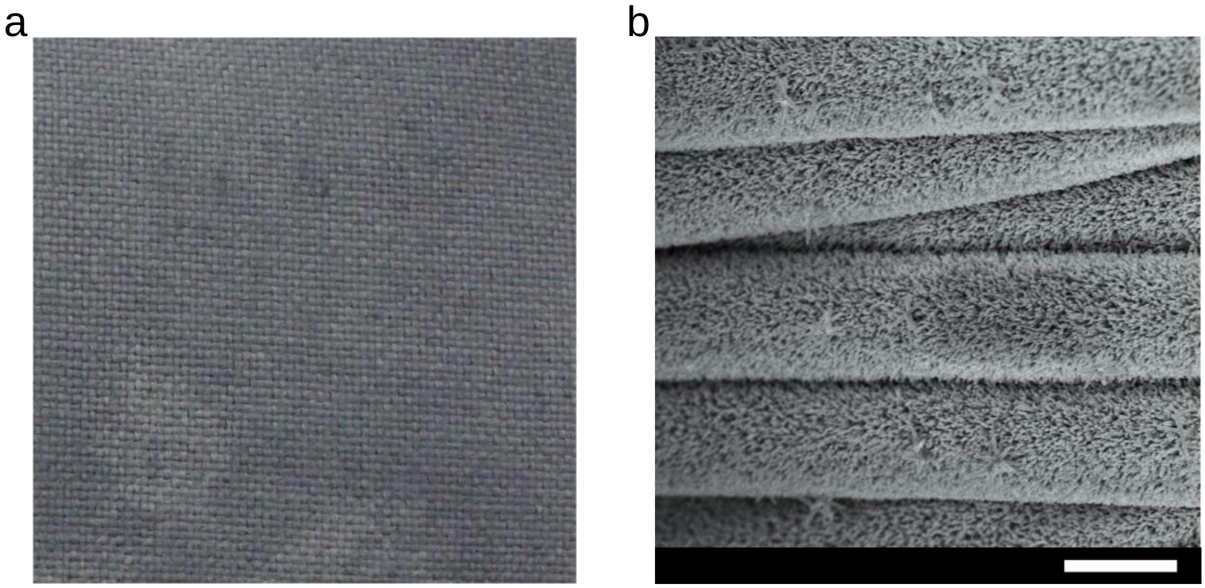

Supplementary Figure 8 | Optical photograph and SEM image. (**a**) Optical photograph and (b) SEM image of the TiO_2 NAs/CT cathode after twisting 1000 cycles.

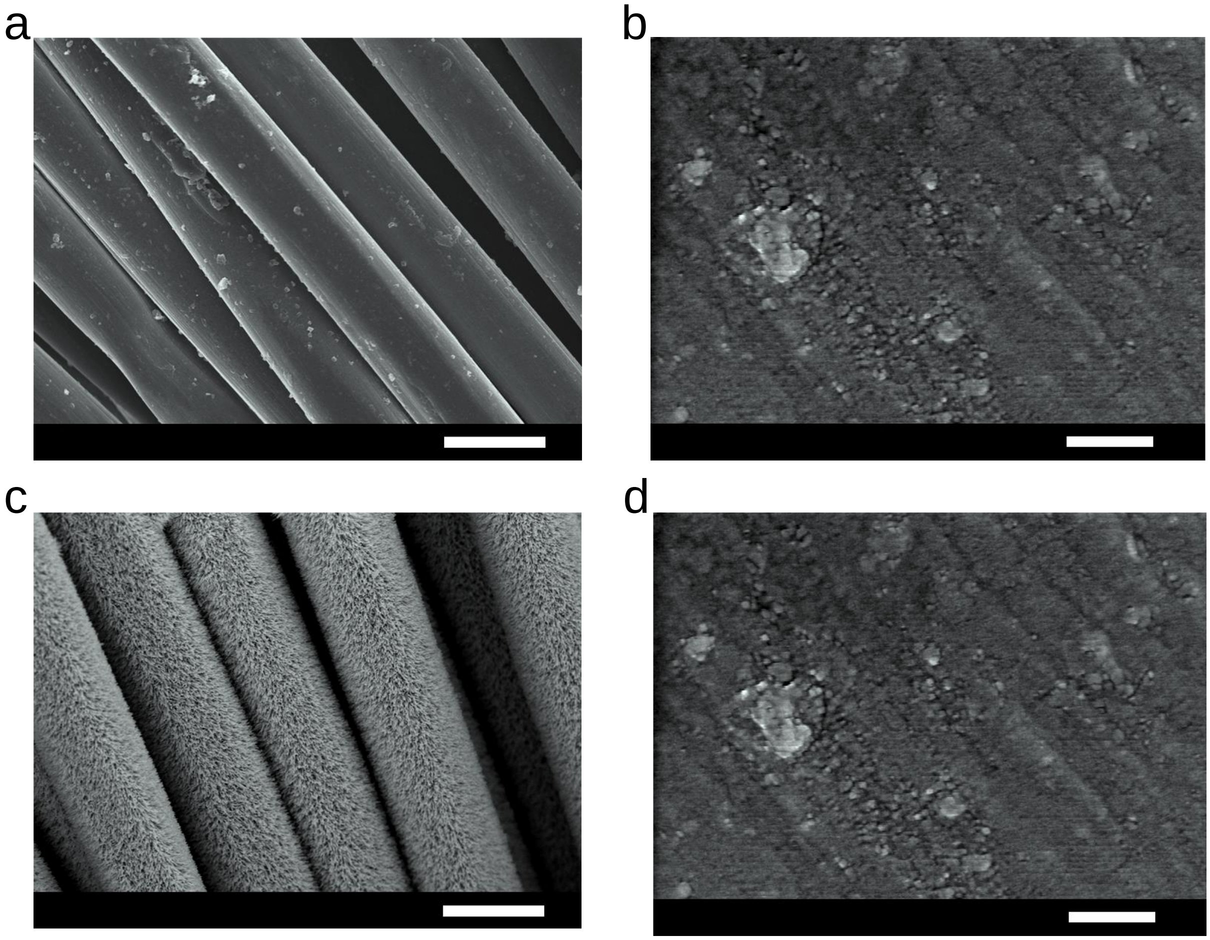

Supplementary Figure 9 | SEM characterization. (**a**) SEM images of the recharged pristine-CT, Scale bars, 10 μm. (**b**) The enlarged image of (**a**). Scale bars, 200 nm. (**c**) Image of the recharged TiO_2 NAs/CT cathode. Scale bars, 10 μm. (**d**) The enlarged image of (**c**). Scale bars, 200 nm. The current density is 100 mA g^{-1} and the specific capacity is 500 mAh g^{-1}.

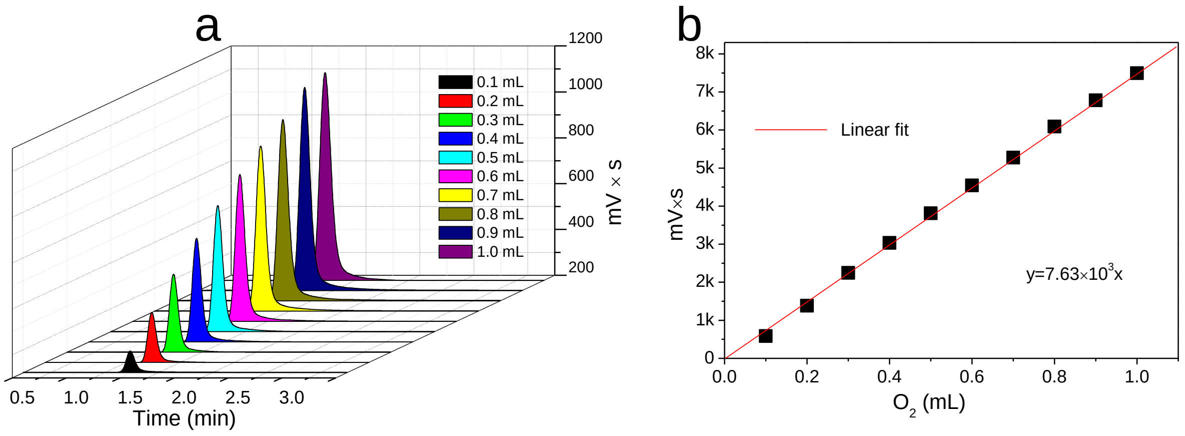

Supplementary Figure 10 | GC signal and calibration curve of O_2. (**a**) GC signal curves obtained by injecting O_2 into column with different volume obtained from a thermal conductivity detector (TCD). (**b**) GC calibration curve obtained according to (**a**).

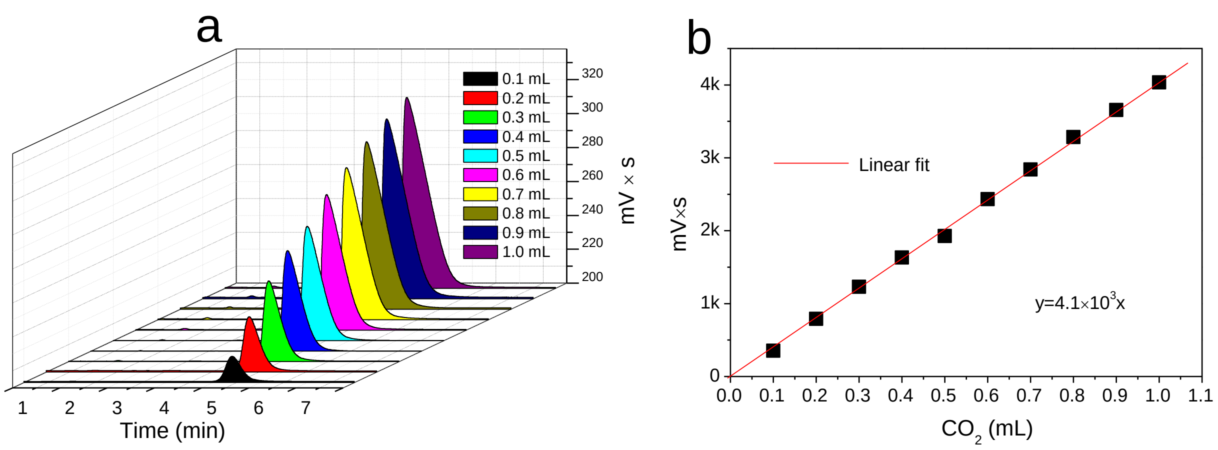

Supplementary Figure 11 | GC signal and calibration curve of CO_2. (**a**) GC signal curves obtained by injecting CO_2 into column with different volume obtained from a thermal conductivity detector (TCD). (**b**) GC calibration curve obtained according to (**a**).

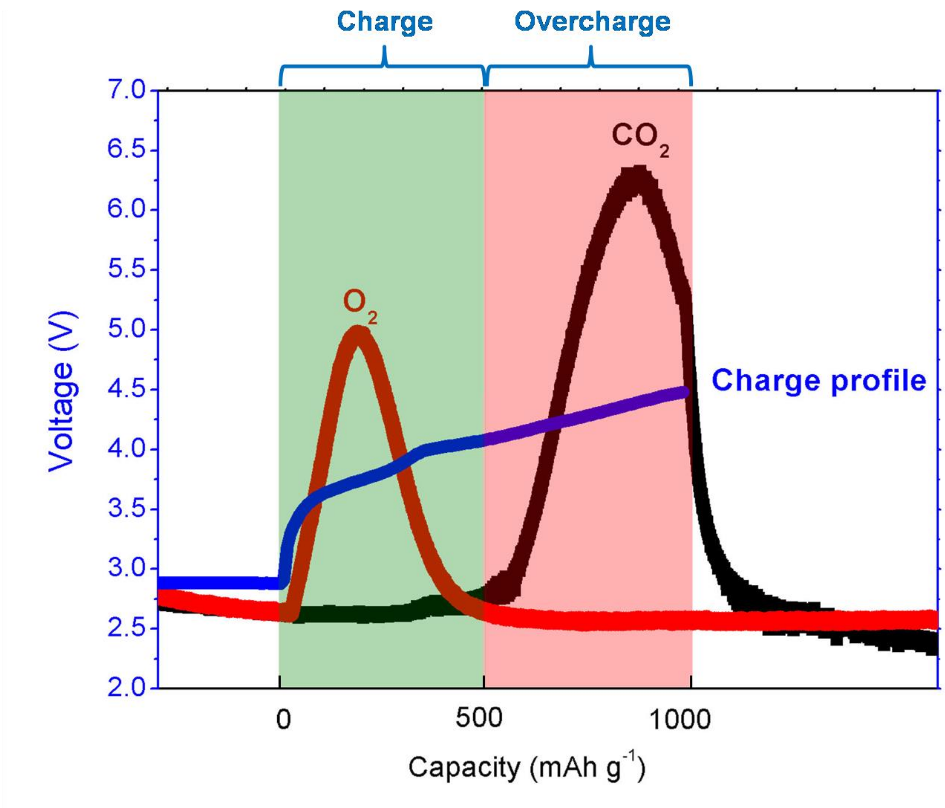

Supplementary Figure 12 | *In-situ* DEMS test. Gas evolution from the cell with TiO_2 NAs/CT cathode, room temperature, with current density of 100 mA g^{-1}.

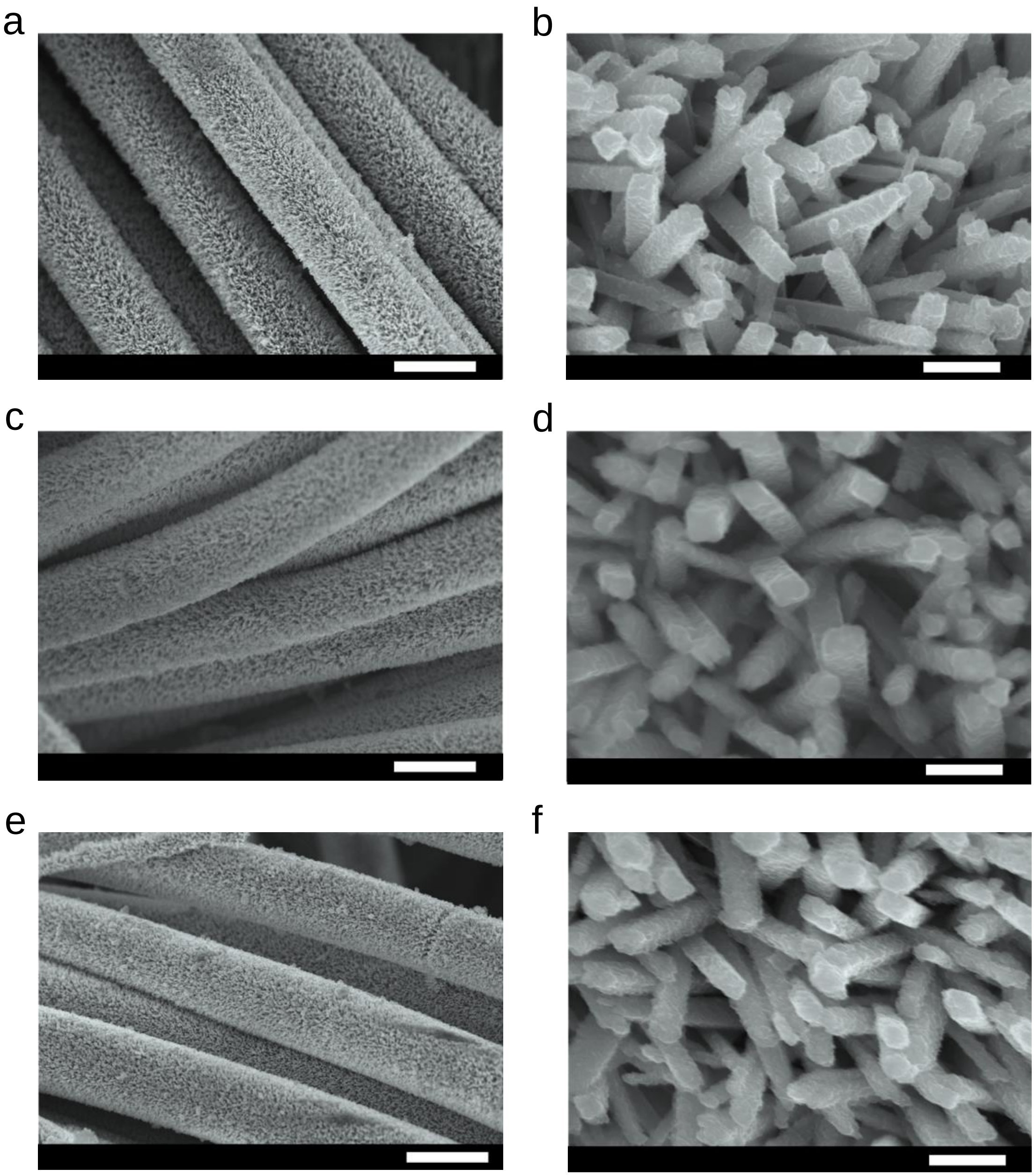

Supplementary Figure 13 | SEM characterization. (**a**) SEM image of the TiO_2 NAs/CT cathode after 10th cycle. Scale bars, 10 μm. (**b**) The enlarged image of (**a**). Scale bars, 500 nm. (**c**) SEM image of the TiO_2 NAs/CT cathode after 50th cycle. Scale bars, 10 μm. (**d**) The enlarged image of (**c**). Scale bars, 500 nm. (**e**) SEM image of the TiO_2 NAs/CT cathode after 100th cycle. Scale bars, 10 μm. (**f**) The enlarged image of (**e**). Scale bars, 500 nm.

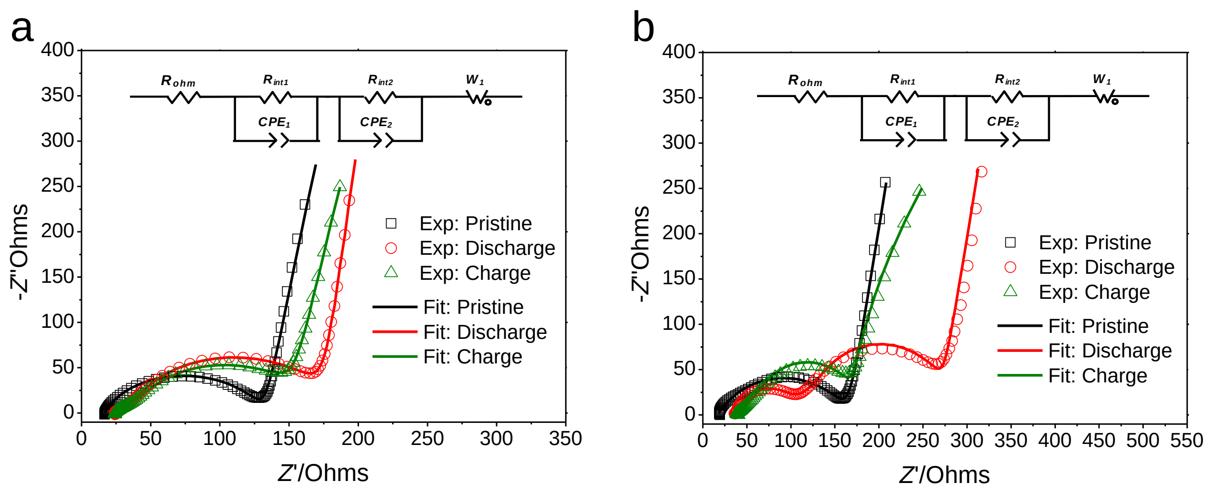

Supplementary Figure 14 | Impedance spectra. (**a**) Electrochemical impedance spectra changes (pristine, first discharge and recharged) of the Li-O_2 cell with pristine-CT cathode. (**b**) Electrochemical impedance spectra changes of the cell with the TiO_2 NAs/CT cathode. The solid line was the fitting line based on the equivalent circuit.

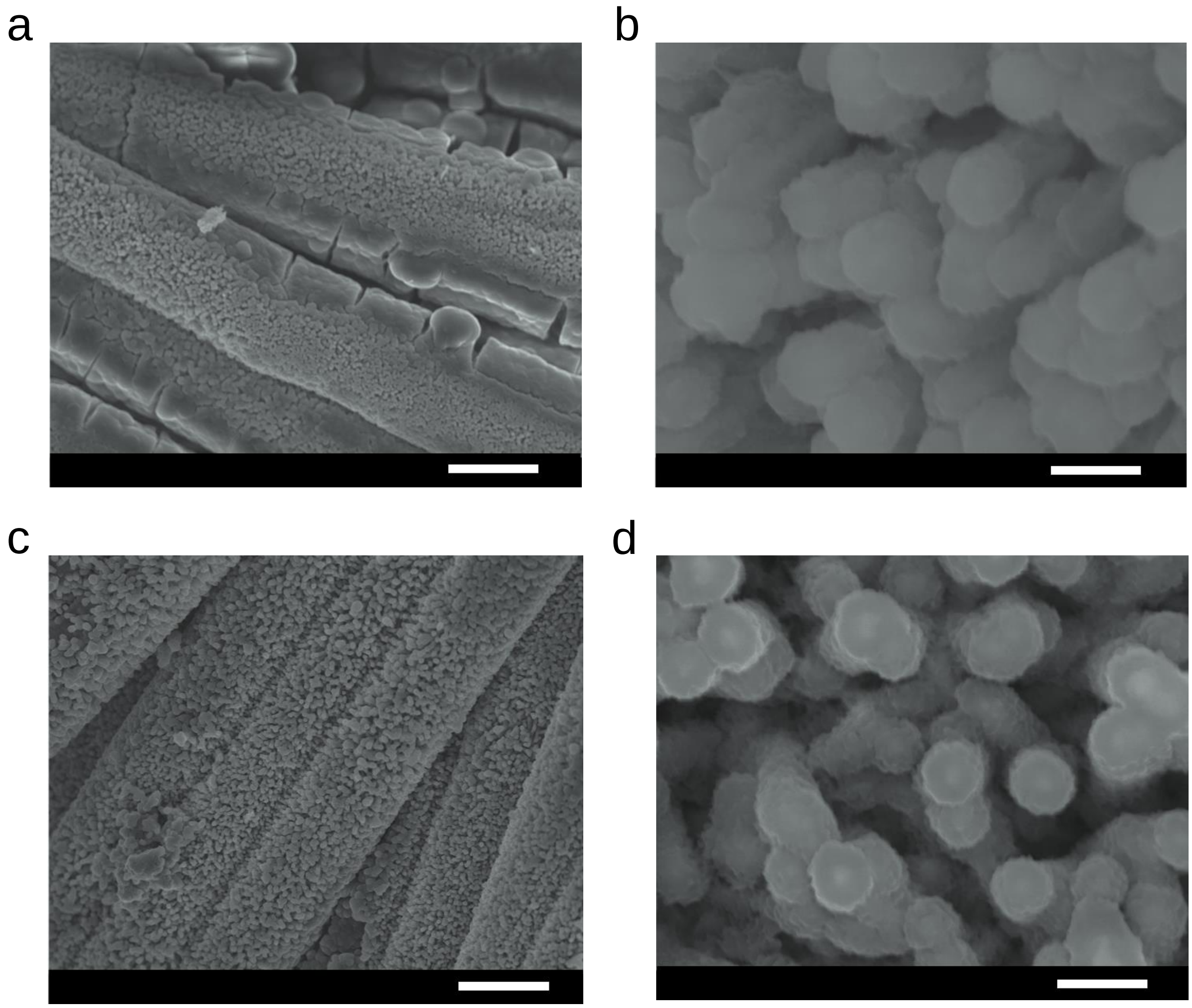

Supplementary Figure 15 | SEM characterization. (**a**) SEM image of the TiO_2 NAs/CT cathode after 356th discharge. Scale bars, 10 μm. (**b**) The enlarged image of (**a**). Scale bars, 500 nm. (**c**) SEM image of the TiO_2 NAs/CT cathode after 356th recharged. Scale bars, 10 μm. (**d**) The enlarged image of (**c**). Scale bars, 500 nm. The current density is 100 mA g^{-1} and the specific capacity is 500 mAh g^{-1}.

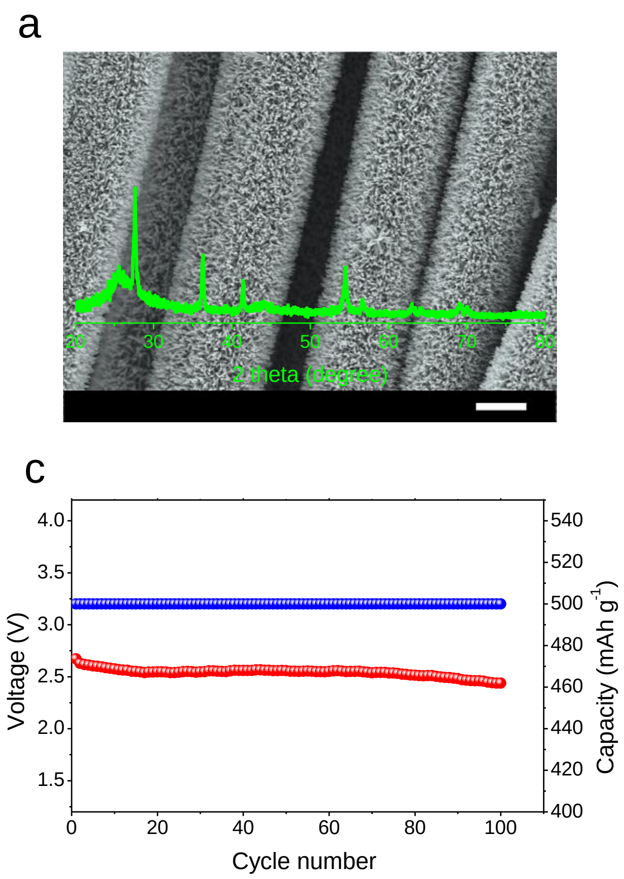

Supplementary Figure 16 | Recoverable performance. (**a**) SEM images of the third recovered TiO_2 NAs/CT cathode. Scale bars, 5 μm. (**b**) SEM images of the fifth recovered TiO_2 NAs/CT cathode. Scale bars, 5 μm. Inset in (**a, b**) is the corresponding XRD patterns. (**c**) The corresponding variation of voltage on the terminal of discharge of the third recovered TiO_2 NAs/CT cathode with current density of 100 mA g^{-1}. (**d**) The corresponding variation of voltage on the terminal of discharge of the fifth recovered TiO_2 NAs/CT cathode.

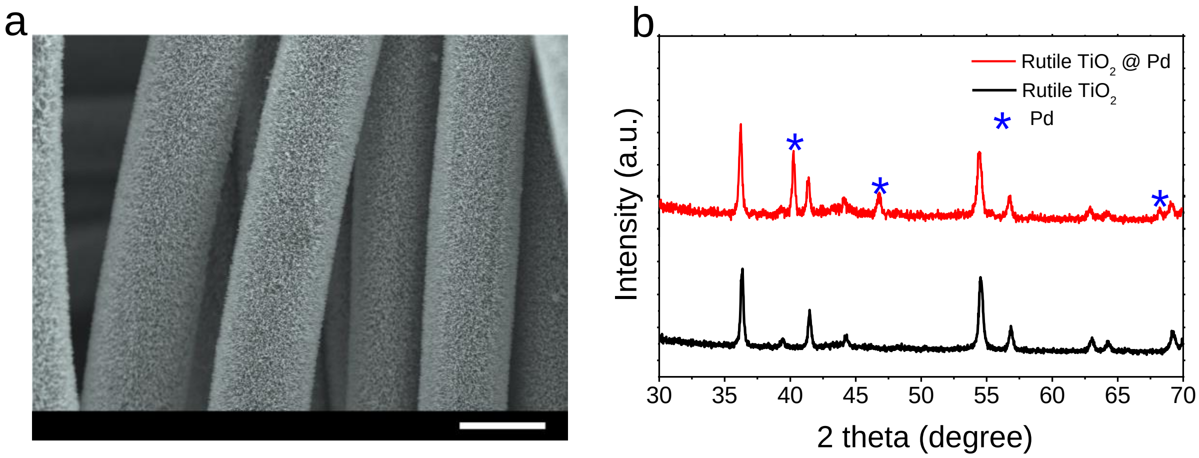

Supplementary Figure 17 | SEM image and XRD patterns. (**a**) SEM image of the Pd modified TiO_2 NAs/CT cathode. Scale bars, 10 μm. (**b**) XRD patterns of the TiO_2 NAs/CT cathode and the Pd modified TiO_2 NAs/CT cathode.

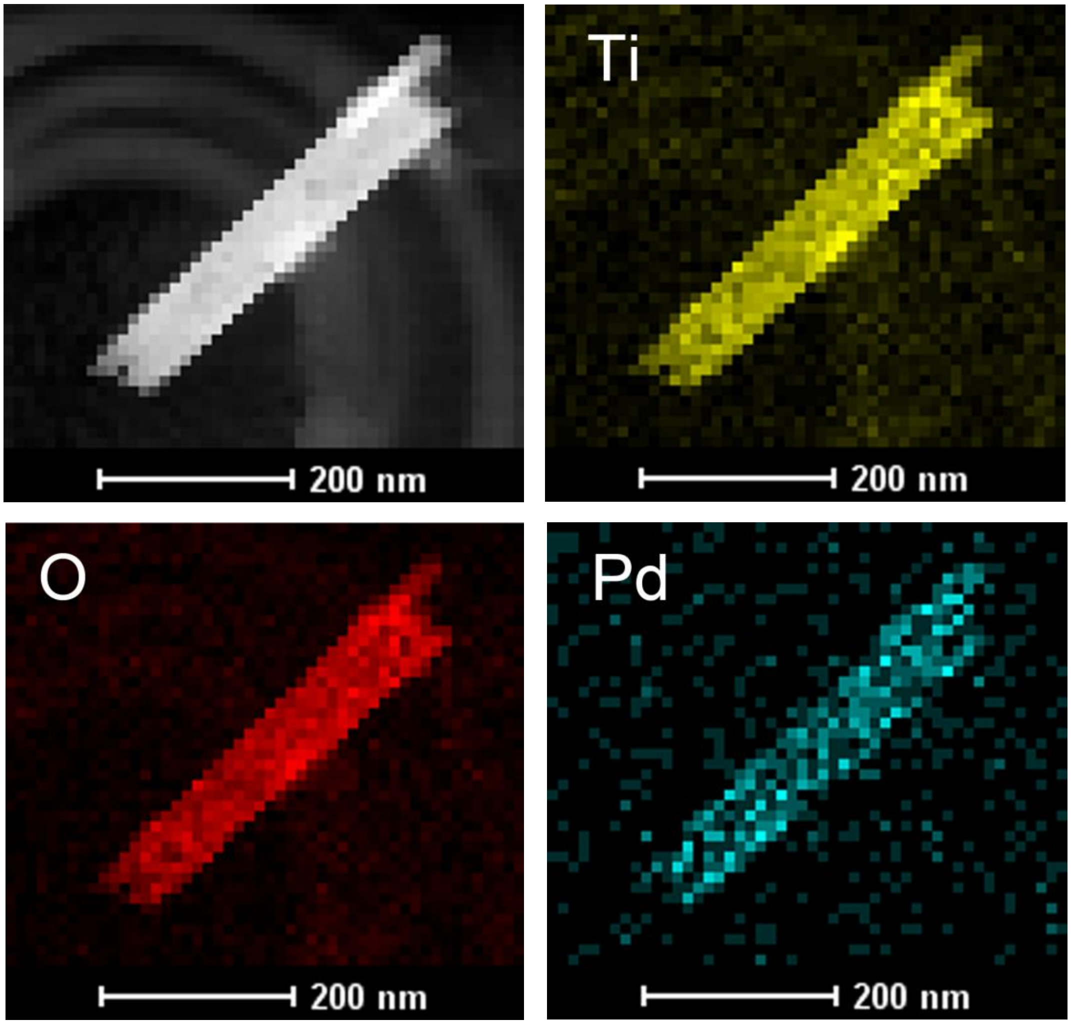

Supplementary Figure 18 | TEM mapping images. TEM image of of the Pd modified TiO_2 NAs/CT cathode and the corresponding elemental mapping images of Ti, O and Pd. The Ti, O and Pd elements are uniformly distributed around a single TiO_2 NAs.

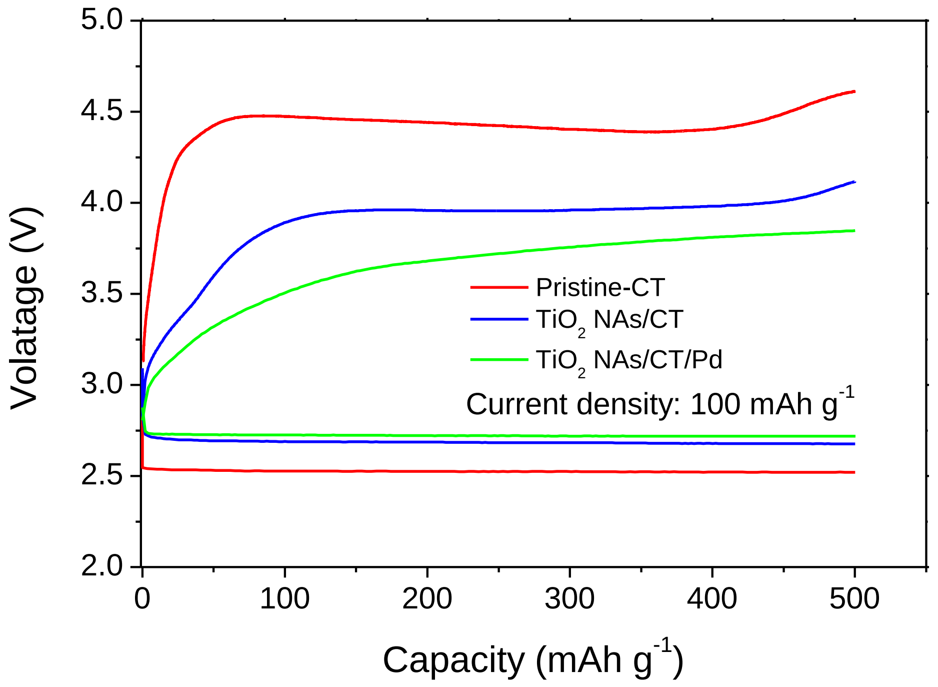

Supplementary Figure 19 | Cell electrochemical performance. First discharge-charge curves of Li-O_2 cells with pristine-CT cathode, TiO_2 NAs/CT, and Pd modified TiO_2 NAs/CT cathodes. The discharge and charge voltage of the Li-O_2 cell can be significantly improved with the help of the Pd modified TiO_2 NAs/CT cathode, which enhances the round-trip efficiency.

Supplementary Table 1 | The amount of the released gas. These results are obtained on the basis of the GC signal spectra and calibration curves (Figs. S10 and S11). Both of two cells with pristine-CT and TiO_2 NAs/CT cathodes were discharged and recharged with the current density of 100 mA g^{-1}.

	Pristine-CT	TiO_2 NAs/CT
mV×s $_{(O2)}$	683.75	716.51
mV×s $_{(CO2)}$	8.71	0.25
$V_{(O2)}$ (μL)	89.61	93.91
$V_{(CO2)}$ (μL)	2.12	0.04

Supplementary References

41. Y. M. Cui, Z. Y. Wen, Y. Liu, A free-standing-type design for cathodes of rechargeable Li-O_2 batteries. *Energy Environ. Sci*. **4**, 4727-4734 (2011).
